# Inducible Expression of *Arabidopsis Response Regulator 22* (*ARR22*), a Type-C *ARR*, in Transgenic *Arabidopsis* Enhances Drought and Freezing Tolerance

**DOI:** 10.1371/journal.pone.0079248

**Published:** 2013-11-14

**Authors:** Na Young Kang, Chuloh Cho, Jungmook Kim

**Affiliations:** Department of Bioenergy Science and Technology and Kumho Life Science Laboratory, Chonnam National University, Buk-Gu, Gwangju, Korea; Iwate University, Japan

## Abstract

The *Arabidopsis* two-component signaling system, which is comprised of sensor histidine kinases, histidine phosphotransfer proteins, and response regulators, mediates cytokinin response as well as various other plant responses including abiotic stress responses. *Arabidopsis response regulators* (*ARR*s) are classified into type-A, -B, and -C. Although the roles of type-A and -B *ARR*s are well established in *Arabidopsis* plant signaling, roles of type-C *ARR*s, *ARR22* and *ARR24*, remain elusive. ARR22, a preferentially cytosolic protein, interacts with certain *Arabidopsis* histidine phosphotransfer proteins (AHPs) and displays phosphatase activity on AHP5. *ARR22* is induced by cold and dehydration. Here, we show that inducible overexpression of ARR22 in *Arabidopsis* enhanced dehydration, drought, and cold tolerance in a dexamethasone-dependent manner, whereas mutation of the putative phospho-accepting Asp to Asn in ARR22 (ARR22^D74N^) abolished these tolerance phenotypes. Overexpression of ARR22 decreased electrolyte leakage in dehydration-, drought-, or cold-stressed transgenic *Arabidopsis* plants compared with that of ARR22^D74N^ or compared with wild-type plants. Transpiration rates and stomatal apertures were not affected by ARR22 overexpression. No significant difference in both dehydration and freezing tolerance was observed between wild-type and *arr22* mutants with or without cytokinin preincubation, consistent with the lack of phenotypes of *arr22* mutants in their vegetative development. Meta-profile analyses of the microarray data on *ARR22*-responsive genes indicate that *ARR22* modulates expression of a variety of abiotic stress-responsive genes, which might contribute to increasing drought and freezing tolerance. Taken together, these results suggest that *ARR22* plays a positive role in the stress tolerance response in part via enhancing cell membrane integrity and that phospho-histidine phosphatase activity of ARR22 may be required for this function.

## Introduction

Cytokinin signaling in *Arabidopsis thaliana* utilizes a multi-step phosphorelay two-component signaling system (TCS) comprised of sensor histidine kinases (AHKs), histidine phosphotransfer proteins (AHPs), and response regulators (ARRs) [1], [2]. CYTOKININ RESPONSE1 (CRE1)/AHK4, AHK2, and AHK3 function as cytokinin receptors and are positive regulators of cytokinin signaling [3], [4], [5], [6], [7]. The three-dimensional structure of the AHK4 sensor domain in complex with cytokinins showed that the membrane-distal PAS domain in the CHASE domain of AHK4 binds cytokinin [8]. AHPs mediate the transfer of phosphoryl groups from AHKs to ARRs [9]. A variety of studies have demonstrated that these AHKs play roles not only in organ growth and development [7], [10], [11] but also in the stress response such as abscisic acid (ABA), drought, cold, and high salinity stress signaling [12], [13], [14]. Five AHPs act as redundant positive regulators of cytokinin signaling [15]. AHP6 is a pseudophosphotransfer protein that acts as an inhibitor of cytokinin signaling for protoxylem formation [16].

ARRs are conventionally classified into either type-A or type-B [9], [17]. The type-B ARRs (ARR1, 2, 10–14, 18–21) are transcription factors that harbor a receiver domain and a large C-terminal region containing a Myb-like DNA-binding domain and a glutamine-rich domain [18], [19] and function as positive regulators of cytokinin signaling [20]. Type-B ARRs directly promote the expression of type-A *ARR*s, and the type-A *ARR*s are rapidly and transiently induced by cytokinin treatment. However, the type-B *ARR*s are not inducible by cytokinins. The type-A ARRs (ARR3-9,15–17) are comprised of a receiver domain and a divergent C-terminal extension and function as partially redundant negative regulators of cytokinin signaling [21], [22], [23], [24]. The negative regulation of cytokinin signaling by the type-A ARRs involves phosphorylation-dependent interactions [25], [26], [27]. ARRs also function in other cellular signaling pathways including the stress response. ARR2 functions in ethylene signal transduction [28]. ARR1 and ARR12 regulate sodium accumulation in the shoots by controlling expression of *AtHKT1;1* which encodes the high-affinity K^+^ transporter in roots [29]. ARR2 induces plant immunity to a bacterial pathogen via TGA1/NPR-dependent salicylic acid signaling [30]. ARR4 interacts with phytochrome B to modulate red light signaling by stabilizing the active Pfr form of phytochrome B, indicating cross-talk between cytokinin signaling and light signaling via a type-A ARR [31].

Recently, type-C ARRs have been defined as ARRs (ARR22 and ARR24), which have a domain structure similar to the type-A ARRs, but their expression is not induced by cytokinins [32], [33], [34]. However, the role of the type-C ARRs in cytokinin signaling is unclear [34]. *ARR22* expression is restricted to the chalaza of developing seeds in transgenic *Arabidopsis* harboring the *ARR22* promoter fused to green fluorescent protein [33], whereas a reverse transcription-polymerase chain reaction (RT-PCR) analysis of various organs demonstrated that the transcripts are predominantly detected in the flowers and siliques as well as in leaves and stems at some level [32]. Ectopic *ARR22* expression in *Arabidopsis* induces dwarf phenotypes and poorly developed roots resembling *wol* cytokinin-receptor mutants with constitutively reduced expression of cytokinin-regulated genes [32], whereas all other type-A *ARR* overexpressors investigated exhibit no significant morphological phenotypes in the absence of exogenous cytokinins [21], [22], [23], [24].

Drought stress is a major threat to crop productivity. Numerous transcription factors and signaling components play roles in the abiotic stress response [35], [36], [37]. The C-repeat-binding factor/dehydration responsive element-binding (CBF/DREB) proteins induce many drought- and cold- inducible genes by binding to the CRT/DRE *cis*-acting element. ABRE-binding proteins/ABA responsive factors (AREBs/ABFs), a group of bZIP transcription factors that recognize an ABA-responsive *cis*-acting element (ABRE), induce the expression of ABA-responsive genes involved in the ABA response and stress tolerance. ABA, a stress hormone, plays a key role in the drought stress response in plants. Intensive research has revealed the molecular mechanism of ABA signal perception and transduction through the pyrabactin resistance 1/regulatory component of ABA receptor1 (PYR1/RCAR1) and the negative regulator protein phosphatase 2C (PP2C) as well as sucrose non-fermenting 1-related protein kinase 2 (SnRK2) [37].

Accumulating evidence has indicated that in addition to ABA, other plant hormones including gibberellins, cytokinins, ethylene, and auxin play roles in the abiotic stress response [12], [13], [38], [39], [40], [41], [42], [43], [44], [45], [46]. In particular, recent studies have revealed the role of cytokinins and the cytokinin TCS in abiotic stress responses such as to cold and drought [12], [13], [38], [41], [44], [45], [47], [48], [49]. AHK2 and AHK3 are involved in mediating the cold signal for the expression of a subset of type-A *ARR*s [13]. *ARR1*, type-B *ARR*, and *AHP1*, *AHP2*, and *AHP3* were also shown to function in *Arabidopsis* cold signaling [47]. AHP2, AHP3, and AHP5 play roles as redundant negative regulators of *Arabidopsis* drought stress response [49]. Cytokinins regulate cold and drought stress responses. A reduction in cytokinins in the roots by root-specific degradation of cytokinins in *Arabidopsis* and tobacco resulted in enhanced root growth and drought tolerance [50]. The *Arabidopsis* cytokinin-deficient *isopentenyl transferase* (*ipt*) mutants displayed enhanced salt and drought tolerance with increasing ABA sensitivity and cell membrane integrity [45]. Enhanced cytokinin synthesis in transgenic tobacco, rice, and peanut under senescence-associated promoter also induced drought tolerance and increased yield [37], [41], [51], [52], [53]. Preincubation of *Arabidopsis* cytokinin signaling mutants and wild-type plants with cytokinin induced enhanced dehydration and freezing tolerance [13], [38], [47]. These observations suggest that increasing cytokinin concentration in plants can promote tolerance against abiotic stresses.

Although the roles of type-A and type-B *ARR*s are well established in *Arabidopsis* cytokinin signaling and cold and drought stress responses, the role of type-C *ARR*s, *ARR22* and *ARR24*, remains elusive. It has previously been reported that ARR22 is a preferentially cytosolic protein and interacts with AHP2, AHP3, and AHP5 [54]. *ARR22* expression is induced by dehydration stress after 1 h and remains at a plateau until 8 h, displaying expression kinetics similar to those of canonical drought-responsive genes, whereas type-A *ARR*s respond to dehydration stress in a transient manner [38]. Drought-responsive expression of *ARR22* occurs in cytokinin-receptor-dependent and receptor-independent pathways [38]. *ARR22* is also expressed in response to 4 h of cold stress [13]. In this study, we addressed the role of *ARR22* by analyzing transgenic *Arabidopsis* expressing *ARR22:HA* in a dexamethasone (DEX) inducible manner in the drought and cold tolerance response. We demonstrated that drought and cold-inducible *ARR22* acts as a positive regulator in dehydration and cold tolerance response, and that a conserved phospho-accepting Asp residue of ARR22 is necessary for conferring stress tolerance to transgenic *Arabidopsis*. Meta-profile and hierarchical analysis of the microarray data on *ARR22*-responsive genes indicate that *ARR22* modulates expression of a variety of abiotic stress-responsive genes which might contribute to increasing drought and freezing tolerance. Our results indicate a novel function of a type-C *ARR* gene, *ARR22*, in the *Arabidopsis* cold and drought stress responses.

## Results

### Generation of Transgenic *Arabidopsis* that Overexpress *ARR22*:*HA* or *ARR22^D74N^*:*HA* in a DEX-Inducible Manner

**Figure 1:**
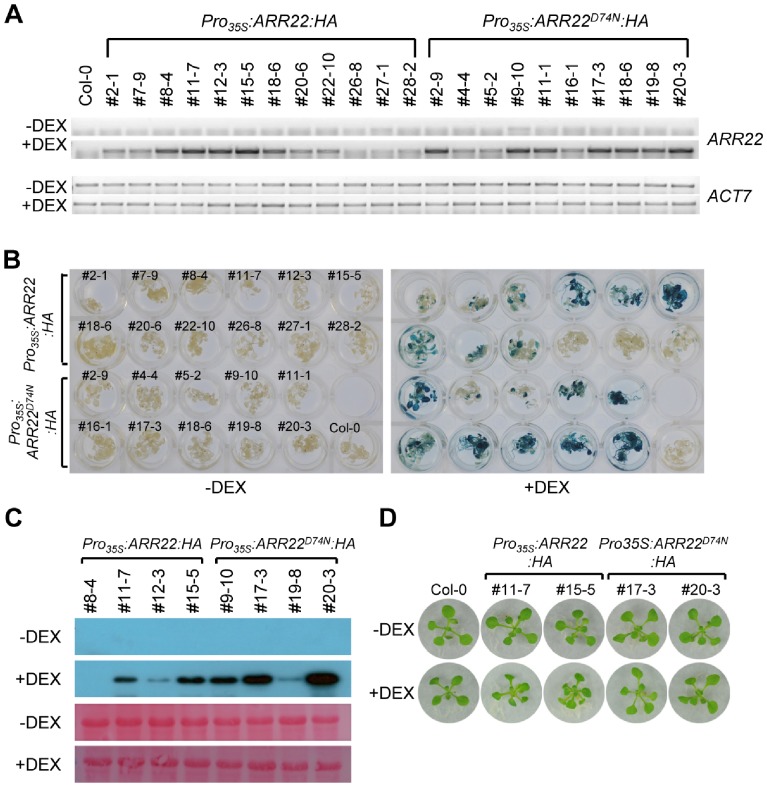
Generation of transgenic *Arabidopsis* displaying DEX-inducible *ARR22* expression. (A) RT-PCR analysis of DEX-induced expression of *ARR22:HA* and *ARR22^D74N^:HA* in *Pro_35S_:ARR22:HA* and *Pro_35S_:ARR22^D74N^:HA* transgenic *Arabidopsis*. Seedlings grown on sterile filter papers on 0.5× MS agar plates for 12 d were transferred to new plates containing 10 µM DEX in 15 ml of 0.5× MS medium and incubated for 6 h in the light at 23°C (+DEX) or mock-treated (-DEX). Total RNAs from each sample were isolated and subjected to RT-PCR analysis for *ARR22*. *ACT7* was employed as the loading control. The numbers on top of the Figure indicate line numbers of transgenic plants. (B) DEX-inducible expression of the *GUS* reporter gene. Seedlings grown on 0.5× MS agar plates for 12 d were incubated in 0.5× MS medium containing 10 µM DEX for 6 h in the light at 23°C (+DEX) or mock-treated (-DEX), followed by GUS staining. (C) Immunoblot analysis of DEX-induced expression of ARR22:HA and ARR22^D74N^:HA. Seedlings grown on 0.5× MS agar plates for 11 d were incubated in 0.5× MS medium containing 10 µM DEX for 48 h in the light at 23°C (+DEX) or mock-treated (-DEX). Total proteins extracted from each sample were subjected to immunoblot analysis with anti-HA antibody. Two lanes shown at the bottom are the protein blots stained with 0.1% Ponceau S in 5% acetic acid. (D) *Pro_35S_:ARR22:HA* and *Pro_35S_:ARR22^D74N^:HA* transgenic *Arabidopsis* plants grown on filter paper prior to dehydration stress. Seedlings were grown for 9 d and incubated with 10 µM DEX or mock for an additional 5 d, then photographed.

*ARR22* expression is induced by dehydration stress, displaying expression kinetics similar to those of canonical drought-responsive genes such as *RD29A*, *RD29B*, and *RD26* [38]. We investigated the potential function of *ARR22* by analyzing the dehydration stress response in transgenic *Arabidopsis* overexpressing *ARR22*. Ectopic expression of *ARR22* in *Arabidopsis* induced dwarf phenotypes and severe developmental arrest such as poorly developed primary roots, and the transgenic seeds were sterile [32]. Thus, we employed a DEX-inducible system [55] to overexpress *ARR22*. We constructed a new vector in which the activator construct that expresses LhGR under the control of the CaMV 35S promoter was combined with a construct harboring *ARR22* and *GUS* under the control of six copies of the *lac* operator to which LhGR binds [56], [57]. This vector allowed *ARR22* expression in a DEX-inducible manner such that the severe developmental arrest and sterility of transgenic *Arabidopsis* constitutively overexpressing *ARR22* was circumvented. Moreover, to monitor the expression of ARR22 proteins, we fused the HA epitope to ARR22 at the C-terminus (*Pro_35S_:ARR22:HA*). The putative phospho-accepting site of ARR22 was predicted from a highly conserved putative phospho-accepting site revealed by amino acid sequence alignment of the type-A ARR proteins [26]. *Pro_35S_:ARR22^D74N^:HA*, in which the putative Asp residue phospho-accepting site at amino acid number 74 was mutated to an inert Asn residue, was also constructed. Transgenic *Arabidopsis* plants harboring *Pro_35S_:ARR22:HA* or *Pro_35S_:ARR22^D74N^:HA* were then generated (Figure 1). Expression of both *ARR22:HA* and *ARR22^D74N^:HA* in various transgenic lines was significantly induced following DEX treatment for 6 h, as shown by the RT-PCR analysis data (Figure 1A). The degree of GUS staining also correlated with the *ARR22* transcript levels (Figure 1A and 1B). An immunoblot analysis was conducted using monoclonal antibody against the HA epitope of the transgenic lines expressing high *ARR22* levels after the 48 h DEX treatment to select the transgenic *Pro_35S_:ARR22:HA* and *Pro_35S_:ARR22^D74N^:HA* lines for the dehydration tolerance test (Figure 1C). We selected two lines, #11-7 and #15-5, for *Pro_35S_:ARR22:HA* and two lines, #17-3 and #20-3, for *Pro_35S_:ARR22^D74N^:HA* that showed significant immunoblot bands. When these plants were grown on filters in 0.5× MS agar plates and treated with DEX for 5 d prior to dehydration stress treatment, they exhibited minimal morphological changes with only slightly epinastic leaves (Figure 1D).

### Overexpression of *ARR22*:*HA* in Transgenic *Arabidopsis* Enhanced Dehydration and Drought Tolerance with Increasing Cell Membrane Integrity in a DEX-Inducible Manner

**Figure 2:**
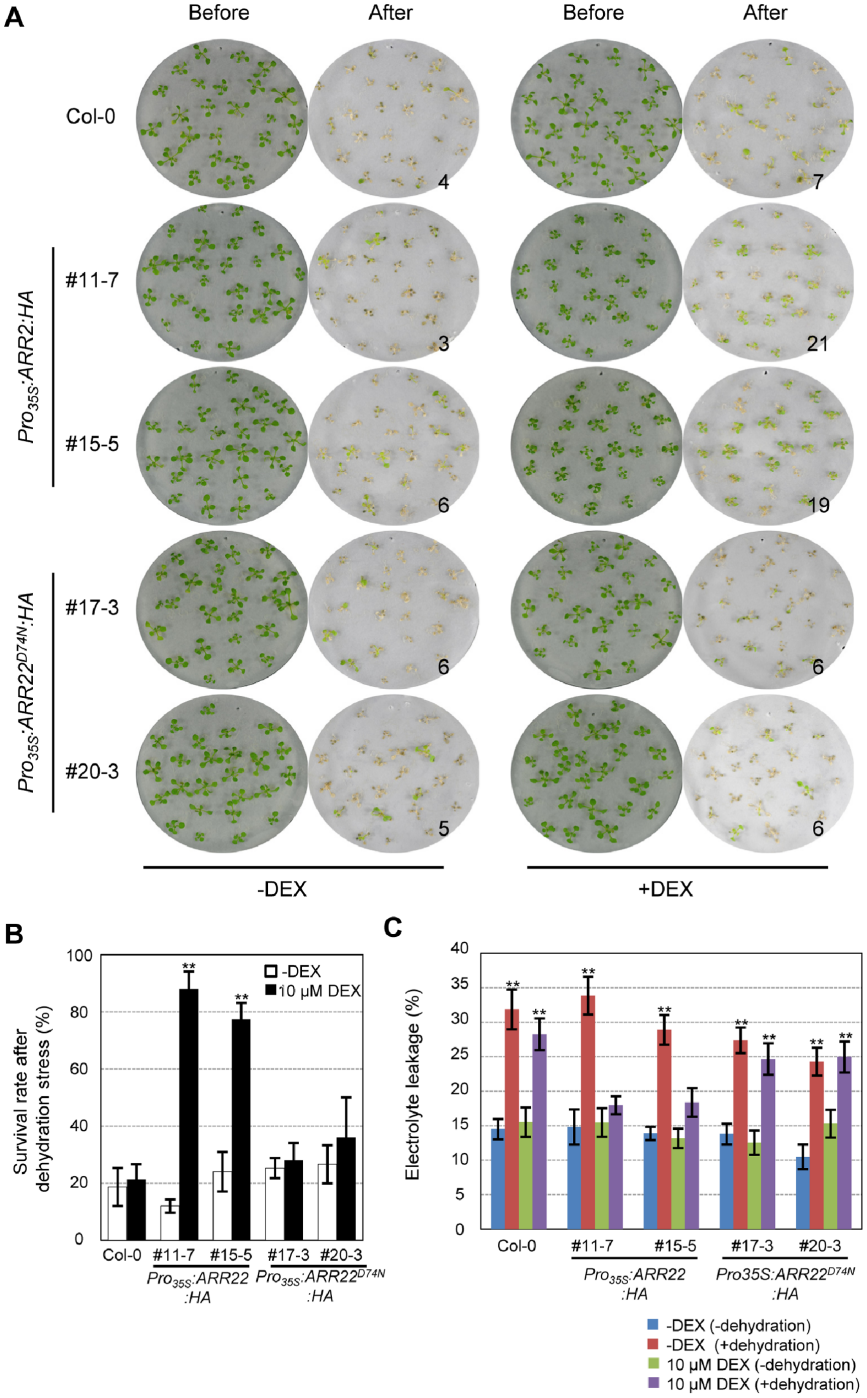
Survival rates and electrolyte leakages of *Pro_35S_:ARR22:HA* and *Pro_35S_:ARR22^D74N^:HA* transgenic *Arabidopsis* plants treated with or without DEX after dehydration stress treatment. (A) Representative filters showing wild-type, *Pro_35S_:ARR22:HA*, and *Pro_35S_:ARR22^D74N^:HA* transgenic *Arabidopsis* plants subjected to dehydration stress tolerance assays. The number indicated beside the filter is the number of plants that survived after recovery process of 25 initial seedlings. Seedlings were grown for 9 d and incubated with 10 µM DEX or mock for an additional 5 d. The DEX or mock-treated plants were dehydrated on filter paper for 3 h and 40 min and allowed to recover with 2 ml of sterile distilled-water for 3 d. Plants surviving after recovery were counted. Experiments were repeated three times. (B) Determination of survival rates after dehydration stress treatment. Experiments are described in the Fig 2A legend. Bar indicate standard error; n = 25. Statistically significant changes compared with plants treated without DEX are indicated by * when *p*<0.05 or by ** when *p*<0.01 (Student’s *t*-test), respectively. (C) Measurements of electrolyte leakage after dehydration stress treatment. Electrolyte leakage was measured for plants treated as described in Figure 2B. Experiments were repeated three times. Bar indicates standard error. Statistical analysis from ten assays was conducted as described in Figure 2B.

We first used the Whatman filter assay method [38] to assess the dehydration tolerance of ARR22:HA-overexpressing *Arabidopsis* plants compared with that of ARR22^D74N^:HA-overexpressing *Arabidopsis* and the wild-type plants. Plants were incubated with or without DEX for 5 d and subjected to dehydration stress on filter paper. As shown in Figure 2A and 2B, ARR22:HA overexpression in transgenic *Arabidopsis* by DEX treatment resulted in significantly higher survival rates under dehydration stress compared with those in wild-type plants. However, transgenic *Arabidopsis* overexpressing ARR22^D74N^:HA showed a similar survival rate to that of the wild-type, even though higher amounts of the ARR22^D74N^:HA protein were expressed in *Pro_35S_:ARR22^D74N^:HA* than ARR22:HA proteins in *Pro_35S_:ARR22:HA* (Figure 1C). This result demonstrated that ARR22 induces dehydration stress tolerance in *Arabidopsis* and that dehydration tolerance acquired by ARR22 overexpression is dependent upon phosphorylation of Asp residue at amino acid 74, a putative phospho-accepting site in ARR22. We measured electrolyte leakage of *Pro_35S_:ARR22:HA* and *Pro_35S_:ARR22^D74N^:HA* transgenic plants that had been incubated with or without DEX compared with that in wild-type to investigate if the increased dehydration tolerance by ARR22 overexpression might be, in part, due to increased membrane integrity. As shown in Figure 2C, dehydration stress increased electrolyte leakage in the wild-type and both mock treated *Pro_35S_:ARR22^D74N^:HA* and *Pro_35S_:ARR22:HA* plants. However, DEX treatment selectively prevented electrolyte leakage in *Pro_35S_:ARR22:HA* plants but not that in wild-type or *Pro_35S_:ARR22^D74N^:HA* plants. These results indicate that ARR22:HA overexpression in *Arabidopsis* increased cell membrane integrity, thereby protecting cell membrane against dehydration-induced membrane injury, and that this increased membrane integrity is dependent upon phospho-histidine phosphatase activity of ARR22.

**Figure 3:**
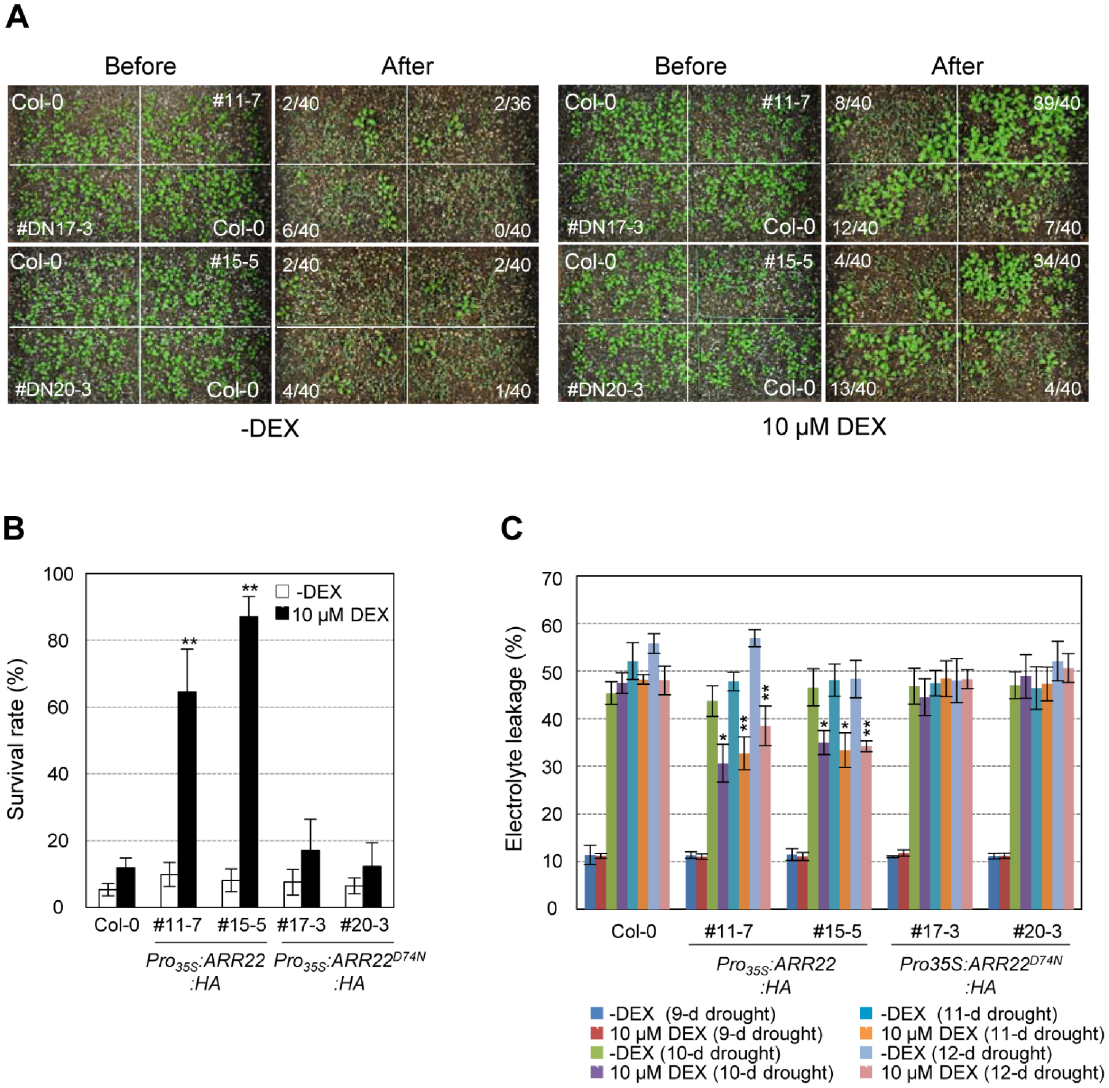
Survival rates and electrolyte leakages of *Pro_35S_:ARR22:HA* and *Pro_35S_:ARR22^D74N^:HA* transgenic *Arabidopsis* plants treated with or without DEX after drought stress treatment. (A) Representative photographs of the survival test under drought stress in plants potted in soil. The number indicated beside the pot is the number of plants that survived the recovery process of the plants tested. Plants, *Pro_35S_:ARR22^D74N^:HA, Pro_35S_:ARR22:HA,* and wild-type plants, were grown for 7 d in soil, treated with DEX or mock for 6 d, and were fully watered once, and then not watered for approximately 3 weeks. The plants were then recovered by watering for 2 d, and the surviving plants were counted. # and #DN indicate the line numbers of the *Pro_35S_:ARR22:HA* and *Pro_35S_:ARR22^D74N^:HA* transgenic plants, respectively. (B) Quantification of plants surviving the drought stress treatment. Experiments were repeated four times. Bar indicates the standard error; n >30. Statistical analysis was conducted as described in Figure 2B. (C) Measurements of electrolyte leakage after drought stress treatment. Plants, *Pro_35S_:ARR22^D74N^:HA, Pro_35S_:ARR22:HA,* and wild-type plants, were grown for 7 d in soil, treated with DEX or mock for 6 d, and were fully watered once, and then not watered for 0, 10, 10, or 12 d. Electrolyte leakage was then measured for the plants treated by drought stress as described above. Experiments were repeated three times. Bar indicates standard error. Statistical analysis from eight assays was conducted as described in Figure 2B.

We also tested drought tolerance of *Pro_35S_:ARR22^D74N^:HA* and *Pro_35S_:ARR22:HA* plants compared with that in the wild-type in pots. As shown in Figure 3A and 3B, the wild-type, *Pro_35S_:ARR22^D74N^:HA*, and *Pro_35S_:ARR22:HA* plants without DEX treatment showed no difference in drought tolerance. However, DEX treatment increased drought tolerance of *Pro_35S_:ARR22:HA* plants greatly but not that of *Pro_35S_:ARR22^D74N^:HA* plants compared with wild-type. Electrolyte leakage was also measured in those plants incubated with or without DEX after the treatment of drought stress for 9, 10, 11, or 12 d. A dramatic increase in electrolyte leakage in all transgenic plants and wild-type was observed after the drought stress treatments for 10 d. Electrolyte leakage in *Pro_35S_:ARR22:HA* plants subjected to the drought stress treatments for 10, 11, or 12 d decreased following DEX treatment but not those of *Pro_35S_:ARR22^D74N^:HA* or wild-type plants, showing that ARR22 overexpression increased membrane integrity of transgenic *Arabidopsis* to enhance drought tolerance (Figure 3C).

### Overexpression of *ARR22*:*HA* in Transgenic *Arabidopsis* Did Not Alter Transpiration Rate and Stomatal Aperture

**Figure 4:**
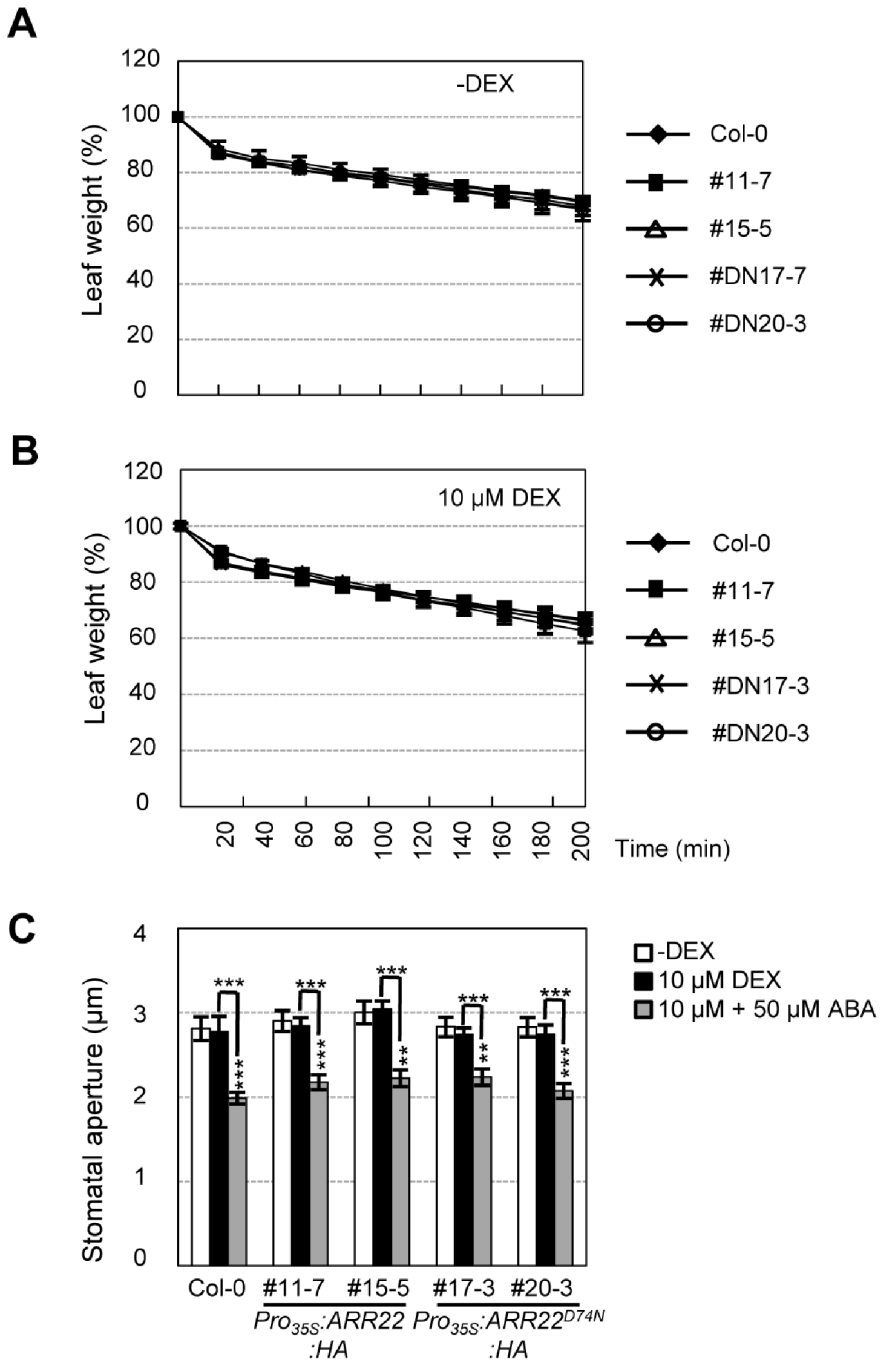
Measurements of leaf weights and stomatal apertures of *Pro_35S_:ARR22:HA* and *Pro_35S_:ARR22^D74N^:HA* transgenic *Arabidopsis* plants treated with or without DEX after dehydration stress treatment. (A) Determination of leaf weights in plants treated without DEX. Experiments were repeated three times. Bar indicates the standard error; n = 10. # and #DN indicate line numbers of *Pro_35S_:ARR22:HA* and *Pro_35S_:ARR22^D74N^:HA* transgenic plants. (B) Determination of leaf weights in plants treated with DEX. Experiments were repeated three times. Bar indicates standard error; n = 10. (C) Measurement of stomatal apertures in plants treated without or with DEX. Experiments were repeated three times. Bar indicates the standard error; n = 20. Statistically significant changes compared with plants treated without ABA treatment are indicated by ** when *p*<0.01 or by *** when *p*<0.001 (Student’s *t*-test), respectively.

The fresh weight loss of detached rosette leaves was measured to test whether enhanced drought tolerance could be attributed, in part, to lower transpiration rates. As shown in Figure 4A and 4B, *Pro_35S_:ARR22:HA* plants showed no difference in fresh weight loss compared with that in wild-type and *Pro_35S_:ARR22^D74N^:HA* plants regardless of DEX treatment. We next tested whether ABA-regulated stomatal opening and closure are altered to confer drought tolerance by ARR22. Stomatal apertures of *Pro_35S_:ARR22:HA*, *Pro_35S_:ARR22^D74N^:HA*, and wild-type plants were measured before and after DEX treatment with or without ABA. DEX treatment of *Pro_35S_:ARR22:HA* plants did not alter ABA promoted-stomatal closure compared with that in *Pro_35S_:ARR22^D74N^:HA* and wild-type plants (Figure 4C). Taken together, these results suggest that the enhanced drought tolerance by ARR22 in transgenic *Arabidopsis* was not attributed to lower transpiration rate or increased stomatal closure.

### *ARR22*:*HA* Overexpression in Transgenic *Arabidopsis* Enhanced Freezing Tolerance in a DEX-Inducible Manner

**Figure 5:**
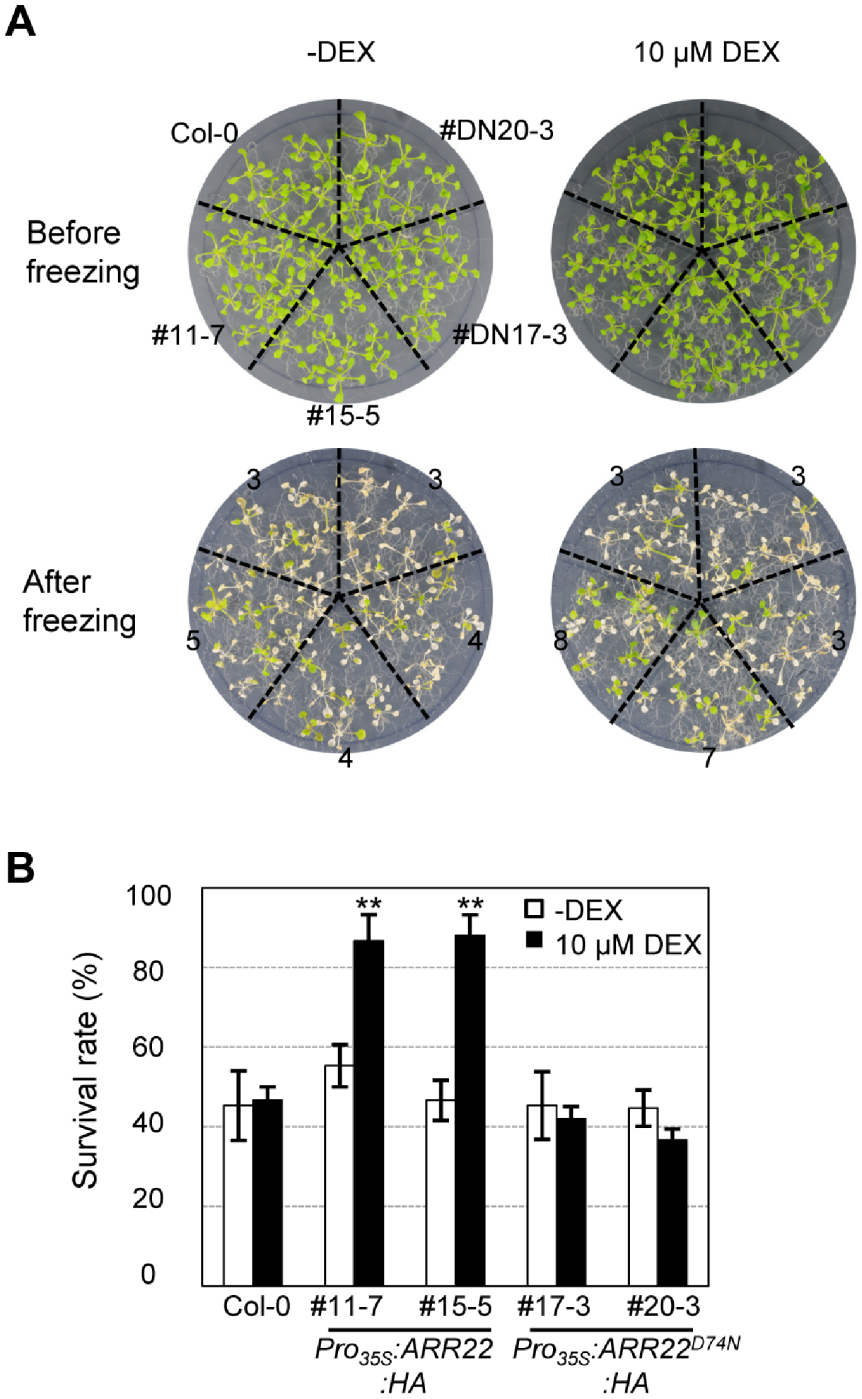
Freezing tolerance assays of *Pro_35S_:ARR22:HA* and *Pro_35S_:ARR22^D74N^:HA* transgenic *Arabidopsis* plants treated with or without DEX. (A) Representative plates showing plants subjected to freezing tolerance assays. Seedlings were grown and treated with DEX as described in Figure 1D. These plants were then treated at −4°C for 4 h and 20 min and photographed after 3 d of incubation at 23°C for recovery. One plate (ø150 mm × 20 mm) contained 10 plants per each plant sample. Three plates were used for each experiment. # and #DN indicate line numbers of *Pro_35S_:ARR22:HA* and *Pro_35S_:ARR22^D74N^:HA* transgenic plants. (B) Plants surviving after freezing stress. Plants were treated as described in Figure 5A. The percentage of plants that survived was calculated. Experiments were conducted five times, and the mean values and standard errors are plotted; n = 30. A statistical analysis was conducted as described in Figure 2B.

It has previously been shown that *ARR22* responds to cold, albeit weaker than drought stress [38]. Moreover, reduced electrolyte leakage in DEX-treated *Pro_35S_:ARR22:HA* plants was observed, suggesting that the enhanced membrane integrity of these plants might contribute to enhanced drought tolerance (Figures 2C and 3C). Stabilization of cell membranes provides tolerance against freeze-induced injury [58], [59]. Thus, we tested freezing tolerance of DEX-treated *Pro_35S_:ARR22:HA* plants compared with that in *Pro_35S_:ARR22^D74N^:HA* and wild-type plants using an *in planta* freezing tolerance assay [13]. As shown in Figure 5, DEX treatment significantly enhanced freezing tolerance of *Pro_35S_:ARR22:HA* plants compared with that in wild-type plants but did not increase freezing tolerance in *Pro_35S_:ARR22^D74N^:HA* plants.

### Effect of Cytokinin Preincubation of Wild-type, *ARR22*:*HA*-overexpressing Plants, and *arr22* Mutants on Dehydration and Freezing Tolerance

**Figure 6:**
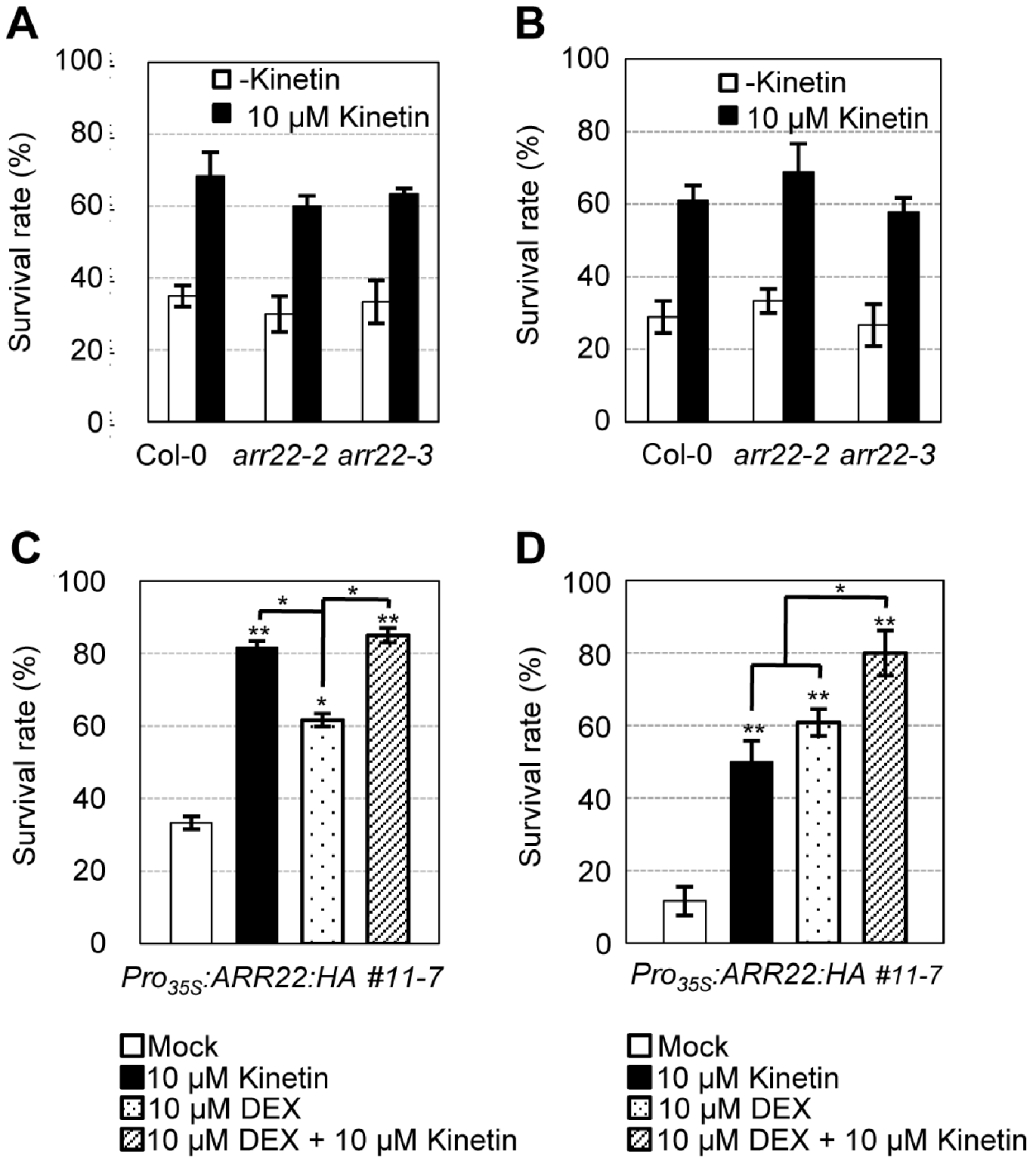
Effect of cytokinin preincubation on drought and freezing tolerance of *arr22*, *Pro_35S_:ARR22:HA,* and the wild-type plants. (A) Dehydration tolerance assays of *arr22* mutants compared with the wild-type plants with or without cytokinin preincubation. Plants were grown for 7 d on Whatman 3MM filter placed on 0.5× MS agar plates, transferred to 0.5× MS agar plates with or without 10 µM kinetin, and grown for an additional 4 d. These plants were dehydrated for 4 h 30 min and recovered by 3 d of rehydration. Surviving plants after recovery were then counted. Mean values and standard errors from triplicate biological replications are plotted. Bars indicate standard errors. n = 20. (B) Freezing tolerance assays of *arr22* mutants compared with the wild-type plants with or without cytokinin preincubation. Plants were grown for 7 d in the light, transferred to MS plates or MS plates containing 10 µM kinetin, and grown for an additional 4 d. These plants were subjected to freezing treatment at −5°C for 4 h. Plants that survived after incubation at 23°C for 3 d for recovery were counted. Experiments were conducted in triplicate, and mean values and standard errors are plotted; n = 30. (C) Dehydration tolerance assays of *Pro_35S_:ARR22:HA* with or without cytokinin preincubation. Plants were grown for 7 d on Whatman 3MM filter placed on 0.5× MS agar plates, transferred to 0.5× MS agar plates with or without 10 µM DEX, and grown for an additional 4 d with or without 10 µM cytokinin treatment. These plants were dehydrated for 5 h and recovered by 3 d of rehydration. Surviving plants after recovery were then counted. Mean values and standard errors from triplicate biological replications are plotted. Bars indicate standard errors. n = 20. (D) Freezing tolerance assays of *Pro_35S_:ARR22:HA* with or without cytokinin preincubation. Plants were grown for 7 d in the light, transferred to 0.5× MS plates with or without 10 µM DEX, and grown for an additional 4 d with or without 10 µM kinetin. These plants were subjected to freezing treatment at −5°C for 4 h. Plants that survived after incubation at 23°C for 3 d for recovery were counted. Experiments were conducted in triplicate, and mean values and standard errors are plotted; n = 30.

*arr22* T-DNA insertion mutant lines, *arr22-2* and *arr22-3*, showed no aberrant phenotype with respect to their vegetative development as well as seed development and nutrition [54]. We also found that those two *arr22* mutant lines did not display any significant difference in dehydration and freezing tolerance compared with that of wild-type (Figure 6A and 6B). Moreover, preincubation of wild-type or *arr22* mutants with cytokinin for 4 d enhanced dehydration tolerance (Figure 6A) and freezing tolerance (Figure 6B) equally. Dehydration tolerance of DEX-treated *Pro_35S_:ARR22:HA* plants preincubated with cytokinin was similar to that of mock-treated *Pro_35S_:ARR22:HA* plants preincubated with cytokinin (Figure 6C). Freezing tolerance of DEX-treated *Pro_35S_:ARR22:HA* plants preincubated with cytokinin was slightly higher than that of DEX-treated *Pro_35S_:ARR22:HA* plants or mock-treated *Pro_35S_:ARR22:HA* plants preincubated with cytokinin but was much lower than the level predicted by combining both freezing tolerance of DEX-treated *Pro_35S_:ARR22:HA* plants and that of cytokinin-treated *Pro_35S_:ARR22:HA* plants (Figure 6D).

### Microarray Analysis of *Pro_35S_*:*ARR22*:*HA* Plants Treated With DEX or Mock

In order to gain insight into how *ARR22* overexpression confers drought and freezing tolerance through changes in gene expression, we conducted a microarray analysis of *Pro_35S_:ARR22:HA* plants treated with DEX compared with a mock treatment using the Affymetrix ATH1 *Arabidopsis* full genome array. We performed this experiment in triplicate followed by statistical analysis to determine the FDRs for a multiple comparison correction to control the type I family-wise error rate [60]. We used two criteria: a fold-change greater than 2.0 and an FDR cutoff of 0.15, to display the genes differentially regulated by ARR22:HA. Fifty-six and 69 genes were upregulated and downregulated by *ARR22* overexpression, respectively. These differentially regulated genes were then classified into 12 functional groups using GO annotation with some manual modifications to gain insight into the function of these genes (Tables S1 and S2) [61]. The functional classification of these genes showed that *ARR22* overexpression caused gene expression changes in a broad spectrum of functional genes. Eighty eight fold induction of *ARR22* was noted, confirming the validity of the microarray data. Unusually high levels of upregulated pathogenesis-related protein 1 (PR1) precursor and peroxidase (22- and 16-fold, respectively) were found (Table S1). Three additional perxoidases were also upregulated, and three MYB transcription factor genes were upregulated. Eight transporter genes are upregulated. In contrast, four expansin genes, which are involved in cell-wall remodeling during plant growth and development [62], were downregulated (Table S2). The *PR2* gene, encoding beta-1,3-glucanase, was downregulated 7-fold. Type-A *ARR*s, *ARR4*, *ARR6*, *ARR7*, and *ARR16* were downregulated over 2-fold, and *ARR5* and *ARR9* were downregulated 1.9- and 1.5-fold, respectively.

**Figure 7:**
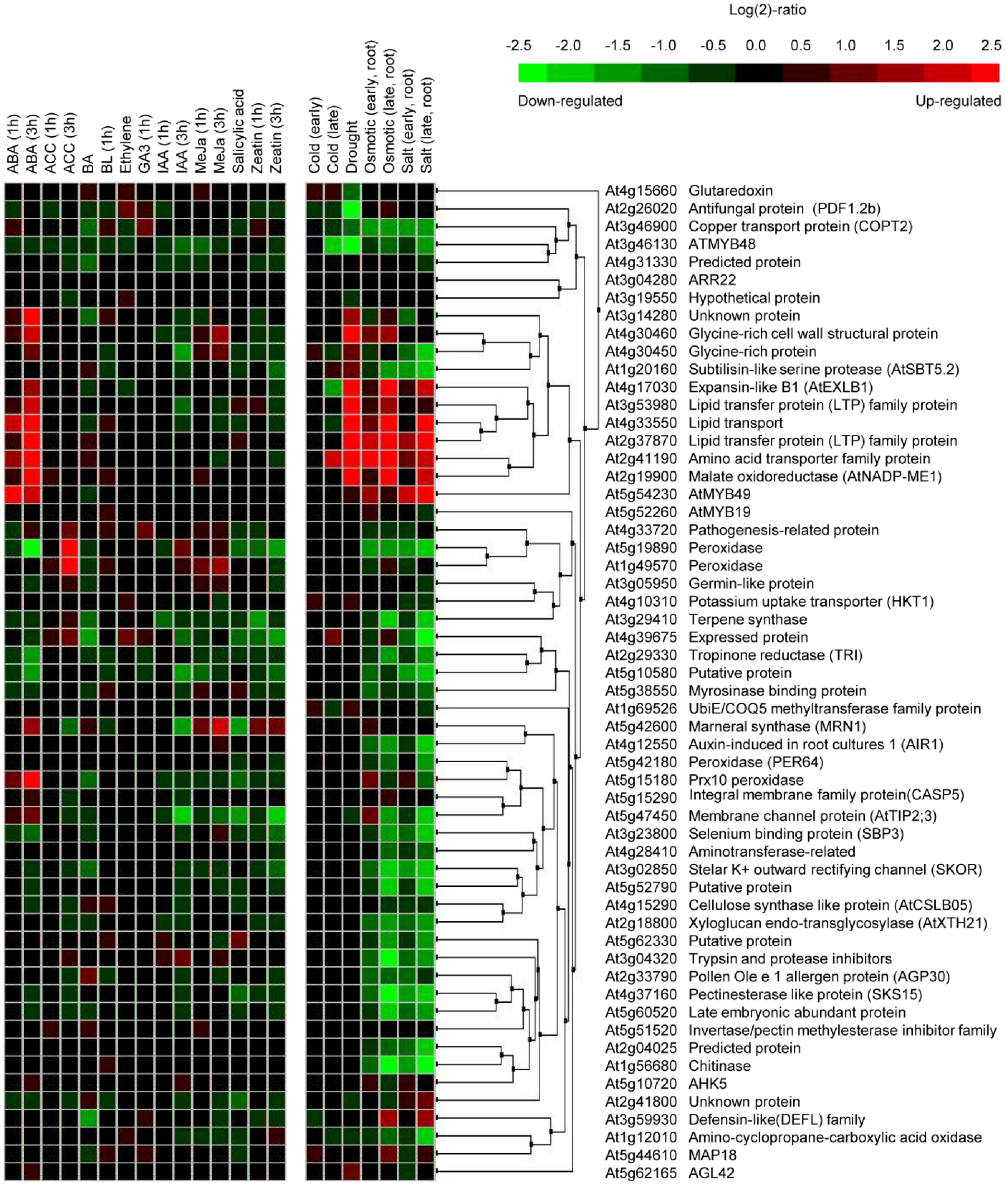
Hierarchical cluster analysis of upregulated genes by *ARR22* overexpression compared with that of the wild-type in response to various stimuli. Genes with *FDR* <0.15 were extracted. Fifty-six genes upregulated with the fold-change >2 were selected. The response of these genes to a given stimulus was obtained from Genevestigator. ABA, abscisic acid; ACC, 1-aminocyclopropane-1-carboxylic acid; BA, 6-benzylaminopurine; BL, brassinolide; GA, gibberellic acid; IAA, indole 3-acetic acid; MeJa, methyl jasmonate; SA, salicylic acid.

**Figure 8:**
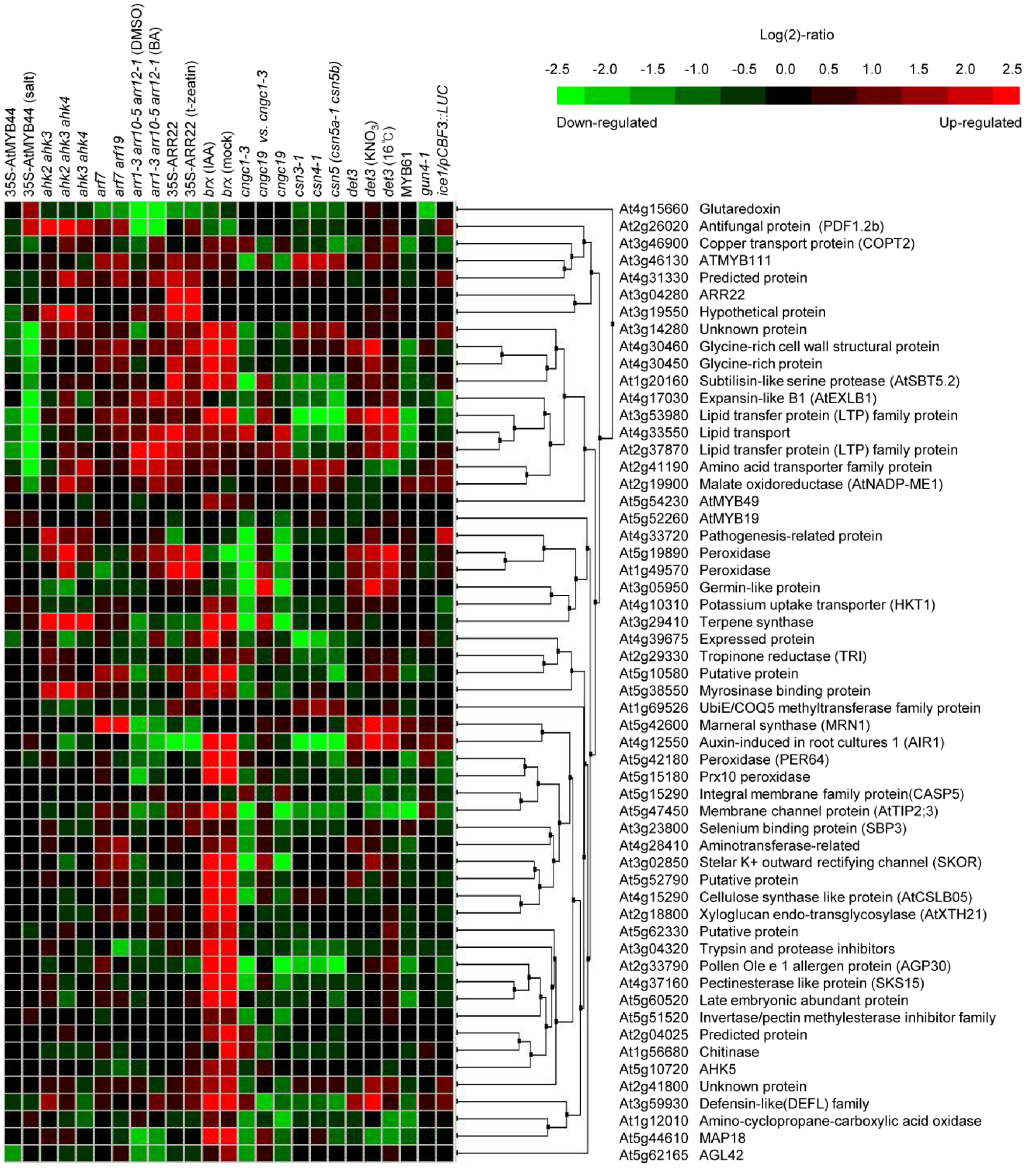
Hierarchical cluster analysis of upregulated genes by *ARR22* overexpression compared with that of the wild-type in a variety of mutants and transgenic *Arabidopsis*. Genes with *FDR* <0.15 were extracted. Fifty-six genes upregulated with the fold-change >2 were selected. *ahk*, *Arabidopsis histidine kinase*; *arf*, *auxin responsive factor*; *arr*, *Arabidopsis response regulator*; *brx*, *brevis radix*; *cngc*, *cyclic nucleotide gated channel*; *csn*, *COP9 signalosome*; *det*, *de-etiolated*; *gun*, *genomes uncoupled*; *ice*, *inducer of CBF expression*.

**Figure 9:**
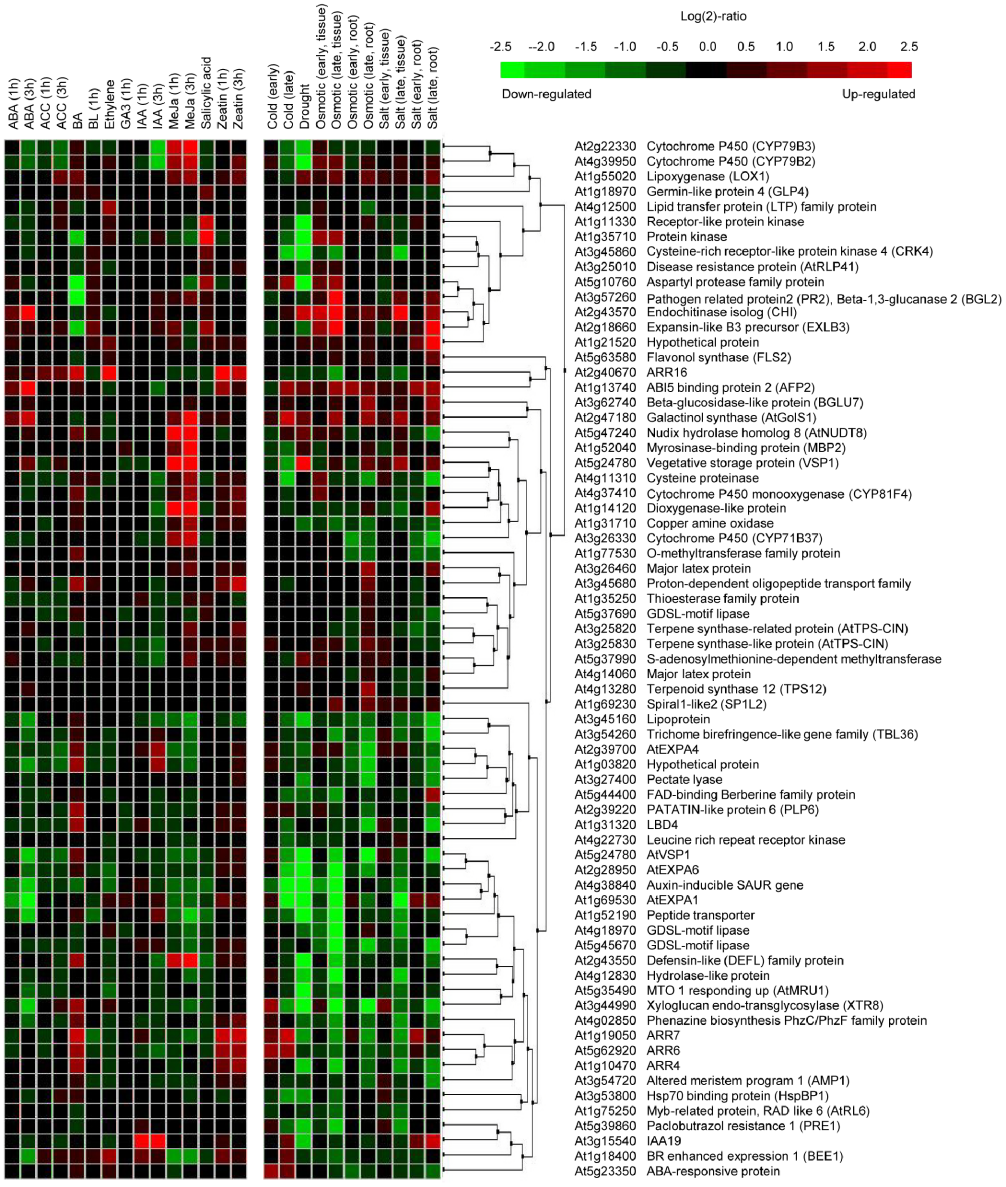
Hierarchical cluster analysis of downregulated genes by *ARR22* overexpression compared with that of the wild-type in response to various stimuli. Genes with *FDR* <0.15 were extracted. Sixty-nine genes downregulated with the fold-change >2 were selected. See Figure 7 for abbreviations.

**Figure 10:**
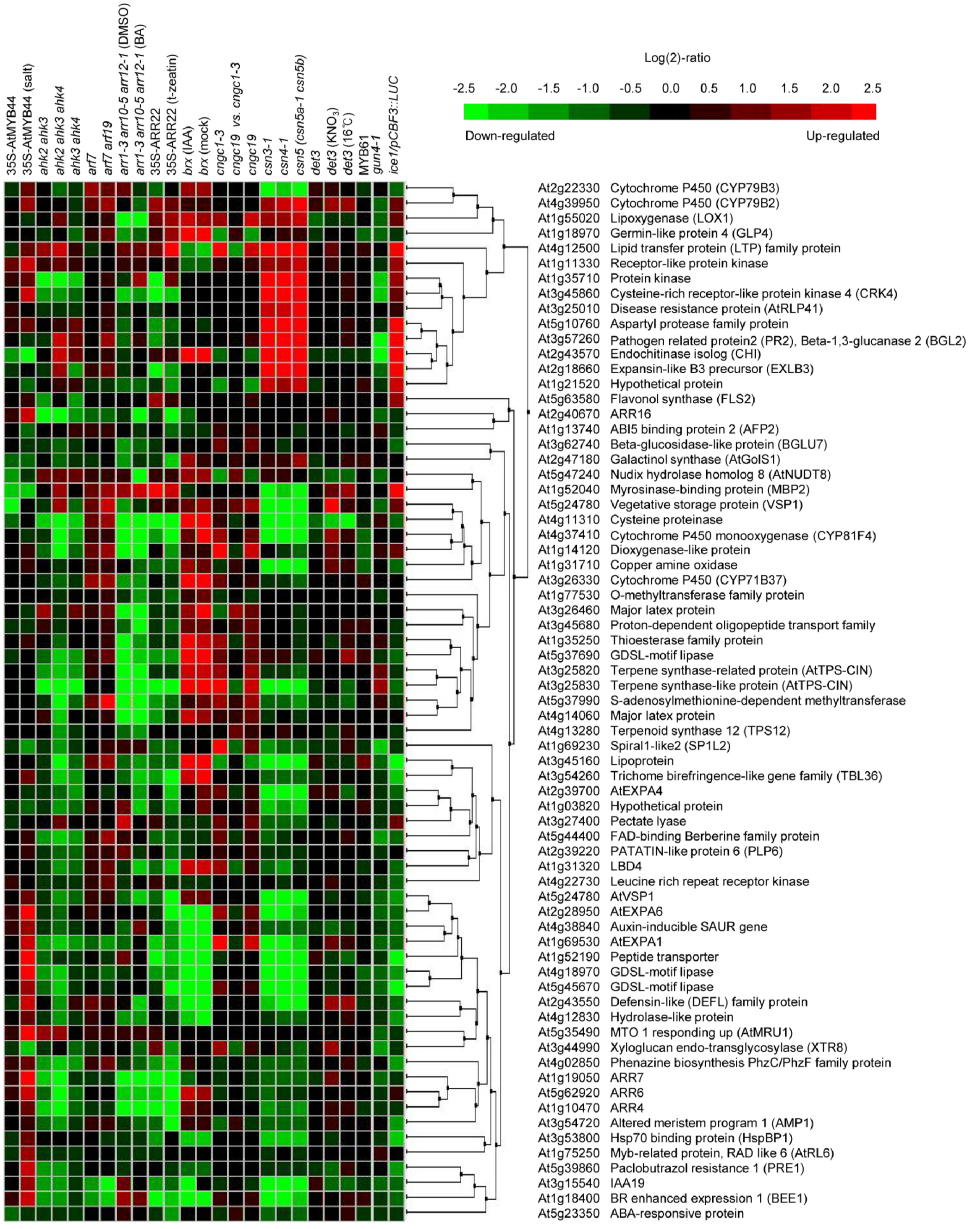
Hierarchical cluster analysis of upregulated genes by *ARR22* overexpression compared with that of the wild-type in a variety of mutants and transgenic *Arabidopsis*. Genes with *FDR* <0.15 were extracted. Sixty-nine genes upregulated with the fold-change >2 were selected. See Figure 9 for abbreviations.

We summarized expression levels according to biological contexts of the samples using the perturbation tool of the meta-profile analysis, which provides a summary of gene expression responses to a variety of plant hormones, abiotic stresses, or mutations, to analyze the expression profiling of genes responsive to overexpression of *ARR22* (Figures 7–10). We first conducted a meta-profile analysis of genes upregulated by *ARR22* overexpression compared with that of mock treatment, followed by hierarchical cluster analysis to group genes with a common expression pattern using a similarity search. Thirteen of the 56 genes showed an upregulated response to the plant hormone ABA (Figure 7). Consistent with a role of ABA in stress response, expression of all these 13 genes is induced by abiotic stresses such as cold, drought, osmotic stress, and/or salt. The rest of other upregulated genes are mainly downregulated by these abiotic stresses. In contrast, most of the upregulated genes showed a very weak response or no response to other plant hormones except that ACC induces two peroxidase genes (Figure 7). These results indicate that ARR22 modulates expression of abiotic stress-responsive proteins including glycine-rich cell wall structure protein, three lipid transfer proteins, MYB49, maneral synthase (MRN1), peroxidase, and membrane channel protein (AtTIP2;3). A large portion of the upregulated genes are distinctively upregulated in a variety of *Arabidopsis* signaling mutants (Figure 8). A similar set of approximately half of the ARR22-upregulated genes were upregulated in *ahk2 ahk3*, *ahk3 ahk4*, and *ahk2 ahk3 ahk4* mutants. A smaller but significant number of the upregulated genes were upregulated in type-B *arr* triple mutants, *arr1 arr10 arr12*, compared with those in the *ahk* multiple mutants. A majority of the upregulated genes were found to be downregulated in *cyclic nucleotide gated channel* (*cngc*) and *COP9 signalosome* (*csn*) mutants. The most striking alteration in the gene expression profiling was noted in *brevis radix* (*brx*) mutant displaying strong upregulation of many upregulated genes by ARR22 overexpression. *BRX* mediates feedback between brassinosteroid levels and auxin signaling in root growth [63]. These meta-profile analyses indicate that the genes upregulated by ARR22 may be linked to the plant hormone signaling networks and growth and development. Microarray analysis of *Arabidopsis* constitutively overexpressing *ARR22* (*35S-ARR22*) with regard to cytokinin responses has been reported previously with T1 transgenic plants because of their sterility [32]. In the present microarray analysis, approximately half of the upregulated genes were upregulated in this *35S-ARR22* line, showing similarity and difference in gene expression profiling between these two lines.

Expression of a majority of the genes downregulated by *ARR22* was weakly upregulated by cytokinins such as benzyladenine (BA) and zeatin (Figure 9), consistent with the previous report [32]. A few sets of the 13 genes were strongly induced by methyl jasmonate. More than two thirds of the downregulated genes are downregulated by abiotic stresses. These results also indicate that *ARR22* is involved in the regulation of abiotic stress-related genes. In the case of *Arabidopsis* signaling mutants, most of the downregulated genes were downregulated except for *arf* and *brx* mutants displaying upregulation of more than half of the genes (Figure 10). A set of the 10 genes were strongly induced in the *csn* mutants. While these gene expression profiling patterns are complex, the meta-profile analyses of the downregulated genes in the signaling mutant backgrounds indicate the link of *ARR22* with plant hormone signaling network and growth and development.

## Discussion

*ARR22*, which is not inducible by cytokinins, responds to dehydration and cold stress [13], [38], indicating a potential role of *ARR22* in the environmental stress response. ARR22 resembles type-A in that it only harbors the receiver domain, but it forms a separate clade along with ARR24 in a phylogenetic tree constructed based on receiver domains but is excluded from both the type-A and type-B ARR family members [32]. Unlike the type-A *ARR*s, *ARR22* does not respond to cytokinins or other plant hormones [32], [33]. Type-C was proposed as a classification for the RRs that have a domain structure similar to the type-A RRs, but their expression is not induced by cytokinins, such as *ARR22* [34]. Here, we show that inducible expression of *ARR22* confers dehydration, drought, and freezing stress tolerance in transgenic *Arabidopsis* plants, in part, by enhancing the increased cell membrane integrity. The meta-profile and hierarchical analysis of the microarray data suggest that ARR22 modulates the expression of abiotic stress-related genes which might contribute to the enhanced drought and freezing tolerance.

We evaluated the role of ARR22 phosphorylation in the stress tolerance response by converting the putative phospho-accepting site, the Asp residue of ARR22 into an inert amino acid, an Asn residue. As mutation in the putative ARR22 phospho-accepting site may significantly reduce the abundance of proteins even at the same mRNA level, we tagged ARR22 with the HA epitope at the C-terminus, allowing us to measure protein levels with an available monoclonal anti-HA antibody. We used transgenic plants that overexpress ARR22^D74N^ proteins at higher levels rather than those of ARR22 proteins to ensure that the decrease in enhanced dehydration tolerance by this mutation that we had predicted was not associated with a smaller amount of ARR22^D74N^ protein expressed in transgenic plants as compared to that of ARR22 proteins. We determined that the Asp residue mutation to Asn at amino acid 74 resulted in complete abolishment of the *ARR22*-overexpression effect on enhancing dehydration, drought, and freezing tolerance as well as on suppressing electrolyte leakage in transgenic plants, thereby indicating that the phospho-histidine phosphatase activity of ARR22 may be critical for protein function in the stress tolerance response. The previous genetic complementation experiments of the *arr22* mutants with a genomic wild-type *ARR22* and *ARR22^D74N^* also highlighted the critical role of the Asp residue at amino acid 74 for conferring a severe growth inhibition phenotype [33].

A previous study demonstrated that when phospho-AHP5 and ARR22 were mixed, the phosphoryl group on AHP5 quickly disappeared but not with ARR22^D74N^ [32]. This result demonstrates that a phosphoryl group on AHP5 might be transiently transferred onto ARR22 but rapidly removed from ARR22, thereby suggesting that ARR22 functions as a phospho-histidine phosphatase on AHP5. ARR22 is preferentially localized in the cytoplasm and interacts with AHP2, AHP3, and AHP5, implying the possible phosphatase function of ARR22 on AHP2 and AHP3, although the phosphatase activity of ARR22 on these AHPs remains to be determined [33]. The meta-profile analysis of gene expression profiling by overexpressing *ARR22* showed that ARR22 antagonized cytokinin-responsive gene regulation. Consistent with this, the genes that were downregulated by *ARR22* overexpression were similarly downregulated by multiple mutations in *AHK* or type-B *ARR* genes. These analyses and proposed phosphatase function of ARR22 on AHP2 and AHP3 indicate that negative regulation of cyokinin-responsive genes by ARR22 overexpression might result from reduced phophoload caused by increased phosphatase activity.

Two *arr22* mutant alleles did not display any phenotype with regard to dehydration and freezing tolerance compared with the wild-type and with or without cytokinin (Figure 6), although *ARR22* overexpression caused an increase in drought and freezing tolerance (Figures 2, 3, and 5). The previous report on *arr22* mutants also showed the lack of any detectable morphological and metabolic phenotypes [33]. Genetic redundancy is not likely, as *arr22 arr24* double mutants showed no apparent phenotype [64]. ARR22 may not be a limiting protein during *Arabidopsis* stress response, but play an auxiliary role in protecting plants against environmental stresses so that the defect in stress tolerance due to the lack of *ARR22* could be complemented by endogenous stress-responsive transcription factors such as CBFs/DREBs. We further found that exogenous treatment of cytokinin BA to *arr22* mutants and wild-type plants caused an enhancement of dehydration and freezing tolerance equally (Figure 6). Stress tolerance of *Pro_35S_:ARR22:HA* plants gained by simultaneous treatment of cytokinin and DEX was much lower than combined stress tolerance of *Pro_35S_:ARR22:HA* plants gained by individual treatment of cytokinin and DEX (Figure 6C and 6D). This result indicates that overexpression of *ARR22* decreases exogenous cytokinin effects of enhancing dehydration and freezing tolerance in *Arabidopsis* probably by increased phosphatase activity of ARR22. As the enhanced stress tolerance can only be detected with *ARR22*-overexpressing plants, it is possible that DEX-induced ectopic expression of *ARR22* may result in a pleiotropic stress that activates an inherent defense response in the plant, which might be contributing to the apparent stress tolerance.

We used *Pro_35S_:ARR22:HA* plants treated with DEX for 5 d for the microarray analysis in this study. As the DEX-treated plants look like the plants grown without DEX treatment (Figure 1D), the microarray data includes the gene expression changes induced by *ARR22*. However, it cannot be ruled out that some altered genes might result from pleiotropic effects due to overexpression of *ARR22* for 5 d. Meta-profile and hierarchical analyses of the genes up or downregulated by *ARR22* overexpression (Figures 7 and 9) indicate that *ARR22* might be involved in the regulation of abiotic stress-responsive genes. Thirteen of the 56 upregulated genes displayed the upregulated response to ABA, and were inducible by abiotic stresses including cold, drought, osmotic stress, and/or salt (Figure 7). The other upregulated genes were downregulated by abiotic stresses. More than two thirds of the downregulated genes were downregulated by abiotic stresses (Figure 9). These analyses indicate that ARR22 modulates the expression of abiotic stress-responsive genes. Proteins encoded by some of these upregulated genes such as lipid transfer proteins, MYB proteins, and MRN1 are related to stress response. A recent study showed that *Arabidopsis lipid transfer protein 3* is involved in plant tolerance against freezing and drought stress and is a direct target of MYB96 transcription factor [65]. ABA-mediated MYB96 activation of cuticular wax biosynthesis has been shown to be involved in drought stress tolerance response [66]. Various studies demonstrated that MYB transcription factors can confer abiotic stress tolerance in transgenic *Arabidopsis* [67], [68], [69], [70]. Triterpenoids are essential precursors for cell membranes and steroid hormones and play roles in plant protection against pathogens and environmental stresses [71], [72], [73]. [74], [75]. MRN1 is involved in the biosynthetic pathway of triterpenoids and plays a critical role in growth and development in *Arabidopsis* [76]. Loss-of-function in *Arabidopsis MRN1* caused various phenotypic changes in plant growth and development and also significantly increased electrolyte leakage compared with that of wild-type [76]. This report indicates that increased *MRN1* expression might contribute to enhanced abiotic stress tolerance in *ARR22*-overepxressing *Arabidopsis* plants. Increased expression of membrane proteins such as integral membrane family protein (CASP5), glycine-rich cell wall structural protein, membrane channel protein (AtTIP2;3) might also contribute to increased cell membrane integrity in *ARR22*-overepxressing *Arabidopsis* plants. A significant downregulation of four expansin genes by *ARR22* was noted (Table S2), indicating that reduced cell elongation and expansion might decrease vulnerability of plants to abiotic stresses.

*PR1* precursor gene was overexpressed 22-fold but *PR2*, encoding beta-1,3-glucanase, was downregulated 7-fold in *ARR22*-overexpressing plants (Tables S1 and S2). Such reciprocal changes in *PR1* and *PR2* expression might contribute to the increase in stress tolerance via reinforcement of plant cell walls [77], [78]. We also discovered overexpression of a peroxidase by 16-fold and that an additional three peroxidases were overexpressed significantly. Peroxidases are involved in removing hydrogen peroxide, a reactive oxygen species (ROS), generated by a variety of stimuli including abiotic stress as part of the stress adaptation response [79], [80], [81]. DELLAs restrain plant growth and promote plant survival during environmental adversity by reducing ROS levels via elevating the expression of genes encoding ROS-detoxifying enzymes [82]. Thus, upregulated expression of peroxidases might contribute to the acquisition of stress tolerance in *ARR22*-overexpressing plants.

## Materials and Methods

### Construction of Transgenic *Arabidopsis* Overexpressing *ARR22*:*HA* or *ARR22^D74N^*:*HA*

DNA fragments coding for ARR22 full-length proteins with the hemagglutinin (HA) epitope tag at the C-terminus were generated by polymerase chain reaction (PCR) using *Pfu* DNA polymerase (Qiagen, Valencia, CA, USA) with the primers 5′-CACC GGATCC ATG GCA ACA AAA TCC ACC GGA-3′ and 5′-GCG GAGCTC TCA AGC GTA GTC TGG GAC GTC GTA TGG GTA AGC ATC GAA GAG GTG GCT AAT-3′. The cDNA used as a template for PCR was synthesized with 5 µg of total RNA isolated from 10-d-old *Arabidopsis* seedlings using SuperScript™ II reverse transcriptase (Invitrogen, Carlsbad, CA, USA) according to the instruction manual. The *ARR22:HA* DNA fragment was inserted into the pENTR™/SD/D-TOPO (Invitrogen) vector. The Asp residue at amino acid 74 of ARR22 was changed to an Asn residue using the QuickChange™ site-directed mutagenesis kit (Stratagene, La Jolla, CA, USA) with the primers, 5′-ATT CTA ATG AAC AAG GAA ATG C-3′ and 5′-G CAT TTC CTT GTT CAT TAG AAT-3′, yielding ARR22^D74N^:HA. The recombination reaction was carried out using Gateway® LR Clonase™ II enzyme mix (Invitrogen) on pENTR™/SD/D-TOPO:*ARR22:HA* or pENTR™/SD/D-TOPO:*ARR22^D74N^:HA* as an entry vector and pB7WG6SLhGR as a destination vector, yielding *Pro_35S_:ARR22:HA* and *Pro_35S_:ARR22^D74N^:HA* constructs, respectively. The pB7WG6SLhGR vector expressing both the LhGR transactivator under the control of CaMV35S and *GUS* as well as the transgene regulated under the Op6 operator was obtained via cloning of the NheI fragment from pOpOff2 (kan) vector [48] into the SpeI site of the pB7WG2 vector [47], allowing DEX-inducible expression of the transgene and *GUS* as a reporter. The *Pro_35S_:ARR22:HA* and *Pro_35S_:ARR22^D74N^:HA* constructs were subsequently introduced into *Arabidopsis thaliana* using the vacuum-infiltration *Agrobacterium*-mediated transformation method, and T3 homozygous transgenic plants were obtained. PCR conditions and primer sequences are shown in Table S3.

### Dehydration, Drought, and Freezing Temperature Treatments

*Arabidopsis* was grown on germination agar plates containing 0.5× Murashige Skoog (MS) medium with vitamins, 1.5% sucrose, 2.5 mM Mes, pH 5.7, and 0.8% agar at 23°C with a 16-h photoperiod and treated essentially as described previously [83]. For dehydration stress, the plants were grown for 7 d on 0.5× MS agar plates, transferred to sterile Whatman 3M filters on 0.5× MS plates, and grown for an additional 5 d or for an additional 4 d with cytokinin treatment. The filters with these plants were moved to empty plates and dehydrated by opening the lid in the light at 23°C. The inner diameter of the plate used was 100 × 15 mm. The dehydrated plants were then rehydrated for 3 d for recovery, and the surviving plants were counted. For the drought stress treatment in the soil environment, plants were grown for 7 d in pots (27 × 17 cm) treated with 10 µM DEX or mock treated for 6 d by spraying the solution once per 2 d, fully watered once, then grown for 3 weeks without watering. The plants were then allowed to recover for 2 d after re-watering, and surviving plants were counted. For freezing tolerance assays, plants were treated with the given freezing temperatures in a temperature-controlled chamber. For the DEX treatment, plants were grown for 7 or 9 d on 0.5× MS agar plates, and then transferred to 0.5× MS agar plates containing 10 µM DEX, and grown for an additional 5 d or for an additional 4 d with cytokinin treatment.

### Electrolyte Leakage Assay

Electrolyte leakage was determined from the rosette leaves of dehydration- or drought-stressed plants. Excised leaflets were placed individually into 50-ml test tubes containing 20 ml deionized water and gently shaken overnight. The percentage of electrolyte leakage was calculated as the percentage of the conductivity prior to autoclaving over that recorded after autoclaving the leaflets. Eight to ten assays were conducted for each sample.

### Measurement of Transpiration Rate

To determine transpiration rates, plants were grown for 10 d and treated with 10 µM DEX or mock treated for a given time by spraying the solution once every 2 d. The weights of detached leaves were measured by weighing freshly harvested leaves placed abaxial side up on a weighing dish. Dishes were kept on a laboratory bench for a given time.

### Measurement of Stomatal Aperture

Plants were grown for 4 weeks in soil with or without 10 µM DEX by spraying the solution once every 2 d. Detached leaves were floated in 30 mM KCl solution (with 1 mM CaCl_2_ and 5 mM MES-KOH, pH 6.15) in the light for 3 h, and the aperture width of stomata was measured [84]. To test the effect of ABA on stomatal closure, detached leaves were incubated in 30 mM KCl solution (with 1 mM CaCl_2_ and 5 mM MES-KOH, pH 6.15) in the light for 3 h at room temperature to ensure stomatal opening. ABA was then added at a 50 µM concentration, followed by incubating the leaves for an additional 3 h. Stomatal apertures of epidermal peels were recorded with a DFC420C camera affixed to a microscope (Leica, DM2500 Microsystems, Wetzlar, Germany) and analyzed by IMAGE-J software (Media Cybernetics, U.S. National Institutes of Health, Bethesda, MD, USA).

### Histochemical GUS Assays

Histochemical assays of GUS activity were conducted by incubating the treated seedlings in 5-bromo-4-chloro-3-indolyl glucuronide (Duchefa Biochemie, Haarlem, The Netherlands) at 37°C for 24 h and removing the chlorophyll from green tissues by incubating them in 100% ethanol, as described previously [85].

### RNA Isolation and RT-PCR

*Arabidopsis* plants were immediately frozen in liquid nitrogen, and stored at −80°C following treatment. Total RNA was isolated from frozen *Arabidopsis* plants using TRI Reagent (Molecular Research Center, Inc., Cincinnati, OH, USA). For RT-PCR analysis of *ARR22*, total RNA was isolated using an RNeasy Plant Mini kit (Qiagen) and subjected to RT-PCR analysis with the primers, 5′- GAGAAAACCAAGTCGATAGAAGTGA-3′ as a forward primer and 5′-CAAGCATCGAAGAGGTGGCTAATG-3′ as a reverse primer, using an Access RT-PCR System (Promega, Madison, WI, USA), according to the manufacturer’s instructions.

### Immunoblot Analysis

Total proteins were extracted from 11-d-old *Arabidopsis* seedlings using standard procedures [86]. Fifty µg of total protein was separated by 12% SDS-PAGE and transferred to Immuno-blot PVDF membranes (Bio-Rad, Hercules, CA, USA), then detected with ECL™ in conjunction with the Western Blotting Detection System (GE Healthcare, Chalfont St. Giles, UK). Monoclonal anti-HA antibody produced in mice (Sigma, St. Louis, MO, USA) was employed as a primary antibody at a 1∶2500 dilution and goat anti-mouse IgG-HRP (Santa Cruz Biotechnology, Inc., Santa Cruz, CA, USA) was used as the secondary antibody at a dilution of 1∶5000.

### Statistical Analysis

Quantitative data were subjected to statistical analysis for every pair-wise comparison, using Student’s *t*-test and statistical software (Predictive Analytics Software for Windows version 17.0 (SAS Institute, Cary, NC, USA).

### Microarray and Statistical Analysis

For Affymetrix GeneChip analysis, *Pro_35S:_ARR22:HA* (line# 11-7) transgenic plants grown for 5 d on MS plates were incubated with mock or with DEX for 5 d, and total RNAs isolated with an RNeasy Plant Mini kit (Qiagen) were subjected to microarray analysis. This experiment was performed in triplicate followed by statistical analysis to determine the false discovery rates (FDRs) for a multiple comparison correction to control for type I family-wise error rate [61]. Five µg of RNA was used to make biotin-labeled cRNA products. The Affymetrix *Arabidopsis* ATH1 genome array GeneChip was used. Probe synthesis from total RNA samples, hybridization, detection, and scanning were performed according to standard protocols (Affymetrix, Santa Clara, CA, USA). Affymetrix GeneChip Microarray Suite version 5.0 software was used to obtain signal values for individual genes. Data files containing probe level intensities were used for quantile normalization by the robust multi-chip average procedure in GenPlex™ version 2.6 software (IS Tech, Whittier, CA, USA) for log_2_ scale transformation [87]. P-values of individual genes were obtained with Welch’s *t*-test [88]. The FDRs for various P-values were determined. A fold-change greater than 2.0 and a FDR cutoff of 0.15 were used to determine the genes differentially regulated by ARR22:HA. We further eliminated the genes that expressed the transcripts showing absent calls in all arrays. Gene function analyses were performed using the High-Throughput GoMiner gene ontology mining software (http://discover.nci.nih.gov/gominer/htgm.jsp). Specification of the many gene annotations was also supplemented by further online database searches such as http://www.arabidopsis.org/tools/bulk/go/index.jsp. Genes were categorized into 12 sub-functional groups using Gene Ontology (GO) annotation with some manual modifications. Meta-profile and hierarchical cluster analyses were performed using Genevestigator. Microarray data were deposited into ArrayExpress with the accession number E-MEXP-3685 at http://www.ebi.ac.uk/at-miamexpress.

## Supporting Information

Table S1 **List of genes upregulated by** ***ARR22*** **overexpression over 2-fold with FDRs <0.15.**
(XLSX)

Table S2 **List of genes downregulated by** ***ARR22*** **overexpression over 2-fold with FDRs <0.15.**
(XLSX)

Table S3 Oligonucleotides and PCR conditions.
(XLSX)
